# The final year for community-dwelling older adults with dementia in an Asian setting: admissions, interventions, and caregiver burden

**DOI:** 10.1093/gerona/glaf227

**Published:** 2025-11-16

**Authors:** Ellie B Andres, Chetna Malhotra, PISCES Study Group, Ellie B Andres, Chetna Malhotra

**Affiliations:** Lien Centre for Palliative Cake, Duke-NUS Medical School, Singapore, Singapore; Lien Centre for Palliative Cake, Duke-NUS Medical School, Singapore, Singapore

**Keywords:** Caregiver, Dementia, End of life, Hospitalization, Palliative care

## Abstract

**Background:**

Current understanding of the last year of life with dementia is disproportionately informed by studies conducted in western contexts, primarily within long-term care settings. This study examines the last year of life experience for community-dwelling older adults and their caregivers in an Asian setting.

**Methods:**

Using prospective longitudinal cohort data from 125 family caregivers to older adults who have died during the study, we estimate separate random effects regression models to identify factors associated with hospital admissions, medical interventions, care experience, and informal caregiving hours. We also estimate costs associated with informal caregiving hours.

**Results:**

Nearly half (48%) of older adults experienced an inpatient admission, and nearly all (92%) experienced a potentially burdensome intervention. Urinary tract infections were the strongest predictor of hospital admissions (adjusted odds ratio [AOR] = 10.42, *p* = .00) and medical interventions (AOR = 9.61, *p* = .02). Pneumonia (AOR = 8.40, *p* = .05) and febrile episodes (AOR = 3.94, *p* = .03) were associated with increased odds of intervention, whereas caregivers who prioritized comfort care only were associated with reduced interventions (AOR = 0.28, *p* = .04) and increased admissions (AOR = 3.20, *p* = .04). Family caregivers provided 42 hours of care per week on average, and 30% gave up their employment to care for the older adult during the older adult’s final year.

**Conclusions:**

Community-dwelling older adults in Singapore experienced similar clinical problems and potentially more burdensome interventions, including feeding tubes and physical restraints, than described previously in other contexts, highlighting the need for a palliative approach not apparent in the frequent acute care utilization, interventions, and caregiving burden observed.

## Introduction

In advanced dementia, a palliative approach focused on improving quality of life through physical, psychosocial, and spiritual support for individuals with dementia and their caregivers is recommended, as endorsed by the World Health Organization Global Action Plan on the Public Health Response to Dementia (2017-2025).^1^ By 2050, the Asia Pacific region is expected to be home to more than half (71 million) of individuals living with dementia.^2^ Yet to date, much of what we know about the end of life (EOL) for older adults with advanced dementia (henceforth “older adults”) and their caregivers—characterized by severe dependency, pneumonia, febrile episodes, infections, eating problems, hospitalizations, and emergency department visits—is disproportionately informed by retrospective studies conducted in long-term care settings in western contexts.^3–6^ In the United States and Europe, nursing homes are the most common place of death for older adults, with those whose proxies are more informed about the dementia prognosis more likely to receive care consistent with a palliative approach, including less potentially burdensome interventions.^7–9^

The EOL experience for older adults and caregivers in Singapore and much of Asia is likely to differ considerably from western contexts, with most older adults living in the community and dying in hospital settings, the burden of care primarily borne by family caregivers with limited long-term care infrastructure, and EOL decisions shaped by cultural beliefs around filial piety, family dynamics, and a distinct absence of palliative approaches.^10–17^ Prior studies comparing Asian and western contexts have confirmed differences in EOL care practices, such as physicians in Asia being less likely to limit life-sustaining treatments at the EOL than their western counterparts and more common use of nasogastric tube feeding at the EOL in Asia.^18–21^

This study aims to examine the last year of life experience of community-dwelling older adults and their caregivers in an Asian setting, exploring factors associated with hospital admissions and medical interventions. Based on prior studies conducted in long-term care settings, we hypothesize that older age, male gender, and medical conditions common at the EOL, such as febrile episodes, pneumonia, and urinary tract infections (UTIs) will be associated with increased hospital admissions and medical interventions.^6^^,^^22^ Based on prior studies in Singapore, we expect caregiver burden, preference for life extension, and the perception that their loved one is suffering will be associated with increased admissions and interventions, whereas caregiver social support will be associated with reduced admissions and interventions.^11^^,^^12^^,^^23^^,^^24^

In secondary analysis, we also explore factors associated with caregiver’s perception of their loved one’s healthcare experience and informal caregiving hours, as well as estimate informal caregiving costs during the older adult’s final year. We hypothesize that caregiver employment and assistance from a live-in domestic helper—typically a woman from a neighboring less-developed country employed to care for the older adult—will be associated with fewer informal caregiving hours. In contrast, caregivers who are spouses or children of the older adult, who co-reside with the older adult, who prefer life extension as a care goal, who provide a high proportion of caregiving for the older adult, and older adults with high functional dependency and frequent agitation behaviors, will be associated with more caregiving hours. Few studies have considered dementia caregiver perceptions of care experience, but a prior study suggested care experience was negatively associated with time providing care, co-residence, and caregiver burden.^25^ Thus, we hypothesize that caregiving hours, older adult co-residence, functional dependency, agitation behaviors, and caregiver anticipatory grief will be associated with worse care experience.

## Methods

### Study design and participants

We use data from the ongoing Panel Study Investigating Status of Cognitively Impaired Elderly in Singapore (PISCES; trial registration: NCT03382223) described previously.^26^ Briefly, we recruited caregivers to community-dwelling older adults from memory clinics and general medicine wards in major public hospitals, home care foundations, and hospices between May 2018 and March 2021. Each recruitment site provided a list of eligible older adults diagnosed with advanced dementia (≥6C Functional Assessment Staging Test) by their physician. Eligible caregivers were family members responsible for treatment decisions and ensuring the older adult’s well-being, who saw them weekly, were aged ≥21 years, and cognitively intact. The study was approved by the Institutional Review Boards of our health system and university (2017/2989), and all participants provided written consent.

### Data collection

Caregivers completed surveys in English, Malay, or Mandarin at baseline and subsequently every 4 months until the older adult’s death, then 8 weeks post-bereavement. The current study draws on preliminary data collected between May 2018 and August 2023 from caregivers to older adults who died during the study. All caregivers who responded to at least one survey (range 1-3) during the older adult’s final year were included.

### Outcomes

The primary outcomes were assessed at each survey, reflecting on the preceding 4 months: *admissions*, defined as whether or not the older adult had an overnight stay in a hospital (yes/no); and *medical interventions* (yes/no: cardiopulmonary resuscitation, tracheal intubation, mechanical ventilation, intensive care unit admission, IV fluids, insulin, oral and IV antibiotics, blood transfusion, oxygen, dialysis, blood thinners, and surgery).

Secondary outcomes were also assessed at each survey: *care experience* measured using the 10-item Satisfaction with Care at the End-of-Life in Dementia (SWC-EOLD) scale in which higher scores indicate greater satisfaction with care^27^; and *informal caregiving hours*, defined as daily hours spent on activities of daily living (ADL), instrumental ADL (IADL), and supervision care, and the number of days spent on these activities in the last month. Based on caregiver hours, we also estimated the monetary value of informal caregiving using the proxy good method, which values caregiving time at the wage rate of a close substitute.^28^ We used the 2023 median monthly gross wage of a full-time healthcare assistant (Singapore Dollars S$2604 [S$1-US$0.77]), based on the Singapore Standard Occupational Classification code.

### Independent variables

Independent variables of interest for analysis of the primary outcomes (admissions and interventions) assessed at each survey included emotional support received from family and friends (1 = strong support; 0 = otherwise); comfort only care goal for the older adult (comfort = 1; 0 = prolong life care goal); older adult acute medical problems (yes = 1/no = 0: for each medical problem: pneumonia, UTI, and febrile episodes); caregiver burden assessed based on the modified 15-item Caregiver Reaction Assessment scale (mCRA)^29^ in which each item was scored on a 5-point Likert scale ranging from strongly disagree (5) to strongly agree (1) and average total scores ranged from 1 to 5 with increasing burden; and caregivers’ perception of whether the older adult was suffering (yes = 1/no = 0), a single item from the Mini-Suffering State Examination (MSSE) developed for end-stage dementia.^30^

Independent variables assessed at baseline included older adult age and gender. Control variables included older adult functional dependency measured using the 7-item Bedford Alzheimer Nursing-Severity Scale (BANS-S)^31^ scored from 1 (no impairment) to 4 (complete impairment); tube feeding use: whether the older adult was currently being fed through a tube inserted through their nose—the primary form of enteral feeding used in Singapore^11^ (yes = 1/no = 0); restraint use: whether the older adult had been physically restrained—a list of different restraint types was described (eg, belts, wrist ties) before caregivers were asked, “Have you or anyone else ever used any of these restraints in caring for the older adult?” yes = 1/no = 0)^12^; caregiver co-residence with the older adult (yes/no); and interventions/admissions when not the dependent variable. Independent variables of interest for analysis of the secondary outcomes (caregiving hours and care experience) included caregiver employment status (employed/not employed); assistance from a domestic helper (yes/no); family relationship (spouse/child/other), caregiver co-residence with the older adult; treatment goal to prolong life; functional dependency (BANS-S); and intensity of behaviors included in the Cohen-Mansfield 14-item agitation inventory, which scores 4 domains based on frequency with higher scores indicating greater intensity: physical/aggressive (eg, kicking), physical/nonaggressive (eg, wandering), verbal/aggressive (eg, threatening), and verbal/nonaggressive (eg, moaning).^32^ For the caregiving hours model, proportion of caregiving contribution (1 = high (≥60%); 0 otherwise) is also included as an independent variable. The care experience model includes caregiver anticipatory grief measured using the 18-item Marwit-Meuser Caregiver Grief Inventory-Short form (range: 18-90; higher values indicate greater grief)^22^ and caregiving hours as independent variables. Control variables for the caregiving hours and care experience models included older adult age, admissions, interventions, use of feeding tubes, and restraints.

### Descriptive variables

Additional variables presented in the descriptive results include older adult ethnicity (Chinese/other), marital status (married/widowed/other), eating dependency (BANS-S item), pain, and whether they appeared malnourished (MSSE items); caregiver age, gender, ethnicity (Chinese/other), marital status (married/never married), education (university/other), housing type (public/other) as most Singaporean households (78%)^33^ reside in public flats, whether they stopped working to care for the older adult (yes/no), whether a healthcare professional had told them how long the older adult was expected to live (yes/no), care experience items from the SWC-EOLD. We collected place of death data from caregivers at 8 weeks post-bereavement.

### Statistical analysis

We describe characteristics of older adults and caregivers using means and proportions per older adult based on available observations in the last year of life.

To assess factors associated with older adult *admissions* and *medical interventions* (primary outcomes), we estimated separate random effects logistic regression models, accounting for the longitudinal panel data. Independent variables of interest include older adult age, gender, febrile episodes, pneumonia, UTIs, comfort-only care goal, family support, and perception of suffering. We control for older adult functional dependency, feeding tube and restraint use, co-residence, and interventions/admissions (when not the dependent variable).

We estimated separate random effects linear regression models to assess our secondary outcomes, *informal caregiving hours* and *caregiver perception of care experience*, accounting for the longitudinal panel data. Independent variables of interest include caregiver employment, domestic helper assistance, relationship (eg, spouse/child), co-residence, comfort-only care goal, anticipatory grief (for care experience), older adult functional dependency, agitation, high caregiving proportion (for caregiving hours), and caregiving hours (care experience). We control for older adult age, admissions, medical interventions, feeding tubes, and restraint use.

We use random effects for each of the 4 analytical models to utilize all available data from repeated measurements over time and maximum likelihood estimation to handle data missing at random.

We calculated the monetary value of informal caregiving time by multiplying mean total informal caregiving hours/week *by* hourly gross wage of a full-time healthcare assistant, then *by* 52 (weeks).

All analyses were conducted in STATA15 (StataCorp LLC).

## Results

### Participant characteristics

To date, more than half (61%, *n* = 132) of older adults from the PISCES cohort have died, among whom 125 had caregivers who completed at least 1 survey (1 survey: *n* = 30; 2: *n* = 33; 3: *n* = 62) during the older adult’s final year (Figure S1). Older adults’ mean age was 86 ± 8 years, 74% were female, 79% were ethnically Chinese, and 70% were widowed (Table 1).

**Figure 1.**
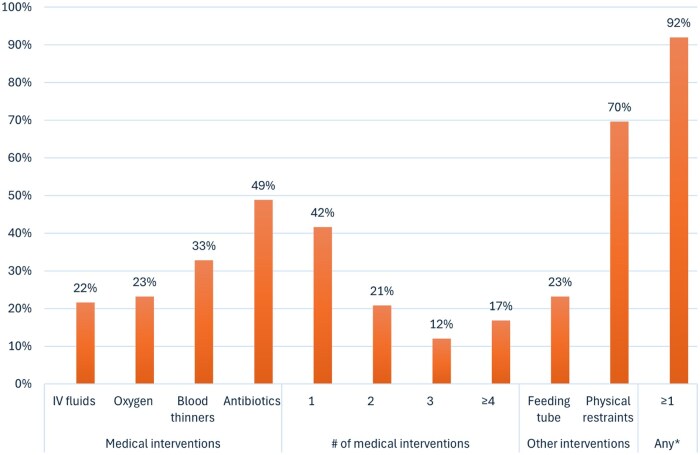
Potentially burdensome interventions experienced by older adults with advanced dementia in their last year of life, *n* = 125. IV, intravenous. *Any intervention includes any medical intervention, feeding tube, or physical restraints.

**Table 1.** Characteristics of older adults and caregivers in older adults’ last year of life (*n* = 125).

Participant	Characteristic	*n*/mean	% / *SD*
*Older adult*			
**Age**	(mean/*SD*)	86	8
**Gender**	Female	92	74%
Ethnicity	Chinese	99	79%
	Other	26	21%
Marital status	Married	33	26%
	Widowed	87	70%
	Other	5	4%
Functional dependency	Score (mean/*SD*); range: 7-28	20.4	3.5
Agitation scores	Physical/aggressive (mean/*SD*); range 1-5	1.3	0.5
	Physical/nonaggressive (mean/*SD*); range 1-5	1.3	0.5
	Verbal/aggressive (mean/*SD*); range 1-5	1.3	0.5
	Verbal/nonaggressive (mean/*SD*); range 1-5	1.7	0.8
*Caregiver*			
Age	(mean/*SD*)	58	10
Gender	Female	92	74%
Co-residence	Live with older adult	94	76%
Relationship	Spouse to older adult	14	11%
	Child of older adult	97	78%
	Other family member	14	11%
Ethnicity	Chinese	98	78%
	Other	27	22%
Marital status	Married	81	65%
	Never married	35	28%
Education	University	30	24%
	Other	95	76%
Employment	Employed	63	50%
	Not employed	62	50%
Housing	Public housing	95	76%
	Other	30	24%

Most caregivers lived with the older adult (*n* = 94; 76%) in public housing (*n* = 95; 76%). Caregivers were primarily female (74%), children (78%) of the older adult, ethnically Chinese (78%), and married (65%) with a mean age of 58 years (median = 60). About a quarter of caregivers (*n* = 30) were university-educated and half (*n* = 63) were employed.

### Medical conditions and symptoms

During the last year of life, most older adults were completely dependent for eating (*n* = 101; 81%), and 42% of caregivers felt the older adult appeared malnourished (*n* = 52). About half of older adults experienced febrile episodes (*n* = 61; 49%), 37% experienced pressure ulcers (*n* = 46), and nearly a quarter contracted pneumonia (*n* = 30; 24%) and UTIs (*n* = 30; 24%), as reported in at least 1 survey during their last year of life. Twelve older adults had a stroke (10%), and 2 had hip fractures (2%). Most had severe functional dependency (mean BANS-S score = 20.4 ± 3.5) as indicated by an average score of ≥3 for all items except one (sleep) on a 4-point scale with 4 indicating complete dependence. Verbal nonaggressive agitation behaviors were the most common agitation behaviors. Many caregivers believed the older adult was suffering (*n* = 60; 48%) and about one-third felt the older adult was in pain (*n* = 43; 34%).

### Healthcare utilization

Although all older adults were living in the community at baseline, 11 (9%) transitioned to a long-term care facility in their last year. Nearly half of older adults (*n* = 60; 48%) experienced at least 1 overnight stay in a hospital. Most older adults were admitted through the emergency department (68%) with the average length of stay spanning 25 days (median = 10, range 1-238 days). Specialist appointments (*n* = 77; 52%) and home healthcare visits by a medical professional (*n* = 83; 66%), including community hospice providers, were also common, whereas 7% of older adults (*n* = 9) received care in the emergency department not resulting in an overnight admission, and 15% attended daycare (*n* = 19). Based on the post-bereavement data collected to date (*n* = 83), half of older adults died at home (*n* = 44, 53%), 35% (*n* = 29) in hospital, 5% (*n* = 4) in nursing homes or long-term care facilities, and 7% (*n* = 6) in inpatient hospice facilities.

### Potentially burdensome interventions

Older adults experienced a range of medical interventions in their final year, including antibiotics (*n* = 61, 49%; oral: *n* = 48, 38%; IV: *n* = 35, 28%), blood thinners (*n* = 41, 33%), oxygen (*n* = 29, 23%), and IV fluids (*n* = 27, 22%) (Figure 1). Forty-two percent received at least one medical intervention (*n* = 52) and 17% (*n* = 21) received 4 or more.

Nearly a quarter of older adults (23%, *n* = 29; *n* = 16 initiated in final year) were tube-fed. Seven in 10 older adults (*n* = 87; *n* = 32 initiated in final year) were physically restrained. Approximately three-quarters (74%, *n* = 93) of older adults experienced feeding tubes, restraints, or both.

In total, 92% of older adults (*n* = 115) received some potentially burdensome intervention in their final year, whether feeding tube, restraints, or other medical intervention.

### Informal caregiving received

Forty-two percent of caregivers (*n* = 53) reported they provided at least 60% of the older adult’s care, and most (82%; *n* = 102) reported a domestic helper assisted with caregiving. Thirty percent (*n* = 37) of caregivers stopped working to care for the older adult during the older adult’s final year. The mean caregiver anticipatory grief score was 52.1 (*SD* = 14.5).

On average, older adults received 30 hours of informal caregiving weekly including ADL and IADL assistance, and 42 hours including supervision, for a median annual cost of S$22 977 (∼US$17 692) and S$32 125 (∼US$24 736), respectively (Table S1).

### Caregiver perception of care experience

Caregivers generally agreed (*n* = 122, 98%) comfort was a primary care goal; however, 31% (*n* = 39) also considered prolonging life a priority. Only 19 caregivers (15%) reported a healthcare provider had provided information about how long the older adult would live. Most caregivers were satisfied with the older adult’s healthcare (*n* = 104, 83% rated it good, very good, or excellent), however, many agreed they would have probably made different decisions regarding the older adult’s treatment and care plan if they had more information (*n* = 78, 62%) and that the older adult needed better medical care (*n* = 58, 46%).

### Factors associated with hospital admissions and medical interventions

UTIs were the strongest predictors of both hospital admissions (AOR = 10.42, *p* = .00) and medical interventions (AOR = 9.61, *p* = .03) (Table 2). Caregivers indicating comfort care only were also associated with increased odds of admission (AOR = 3.20, *p* = .04), but decreased intervention (AOR = 0.28, *p* = .04). Older adults who experienced febrile episodes (AOR = 3.94, *p* = .03) and pneumonia (AOR = 8.40, *p* = .05) were more likely to receive medical interventions.

**Table 2.** Factors associated with older adult inpatient admissions, medical interventions, caregiving hours, and quality of care ratings (*n* = 125).

	Admissions				Medical interventions			
Factors	OR	*p*	[95%	CI]	OR	*p*	[95%	CI]
*Older adult*								
Age	1.05	.24	0.97	1.13	0.98	.65	0.91	1.06
Female	0.37	.10	0.11	1.20	1.21	.76	0.35	4.25
Febrile episode	2.57	.08	0.90	7.37	**3.94**	.03	1.17	13.30
Pneumonia	3.39	.07	0.91	12.72	**8.40**	.05	0.97	72.44
Urinary tract infection	**10.42**	.00	2.69	40.28	**9.61**	.02	1.55	59.65
*Caregiver*								
Comfort only care goal	**3.20**	.04	1.06	9.59	**0.28**	.04	0.08	0.93
Strong family support	0.51	.17	0.20	1.33	0.71	.52	0.25	2.02
Believed older adult was suffering	1.64	.33	0.61	4.43	2.71	.07	0.93	7.93
Burden (mCRA)	1.05	.89	0.53	2.07	1.61	0.24	0.73	3.55
	**Informal caregiving hours**				**Quality of care ratings**			
	Coeff	*p*	[95%	CI]	Coeff	*p*	[95%	CI]
Employed caregiver	**−18.10**	.00	−28.13	−8.08	−0.09	.87	−1.18	0.99
Domestic helper assistance	−3.58	.54	−14.92	7.76	1.12	.07	−0.07	2.31
Spouse of older adult	24.82	.11	−5.87	55.51	**−5.03**	.00	−8.29	−1.76
Child of older adult	13.19	.34	−13.64	40.03	**−5.88**	.00	−8.73	−3.02
Co-residence	**26.84**	.00	16.19	37.50	0.03	.95	−1.14	1.21
Comfort only care goal	−4.22	.41	−14.30	5.86	0.53	.34	−0.56	1.62
Functional dependency	0.05	.95	−1.39	1.48	**−0.20**	.01	−0.36	−0.04
Physical aggressive agitation	5.10	.34	−5.27	15.48	−0.21	.70	−1.29	0.86
Physical nonaggressive agitation	10.71	.06	−0.21	21.63	0.57	.34	−0.60	1.73
Verbal aggressive agitation	6.64	.24	−4.54	17.83	**−1.50**	.01	−2.64	−0.36
Verbal nonaggressive agitation	2.54	.46	−4.24	9.33	0.52	.15	−0.19	1.23
High caregiving proportion (60%+)	**18.57**	.00	8.32	28.82				
Anticipatory grief					**−0.08**	.00	−0.12	−0.04
Caregiving hours					**0.02**	.02	0.00	0.03

The admissions and medical intervention models controlled for dependency (BANS), use of feeding tubes, use of restraints, co-residence, and interventions/admissions when not the dependent variable. The informal caregiving hours and care experience models control for older adult age, admissions, interventions, and use of feeding tubes and restraints.

Abbreviations: OR, odds ratio; Coeff, coefficient; mCRA, modified 15-item Caregiver Reaction Assessment scale.

ORs and coefficients are bolded where significant (*p *≤ .05).

### Factors associated with informal caregiving hours and care experience

Co-residence (β = 26.84, *p* = .00) and high caregiving proportion (β = 18.57, *p* = .00) were strongly associated with caregiving hours, whereas employment (β = −18.10, *p* = .00) was associated with significantly less (Table 2). More caregiving hours (β = 0.02, *p* = .02) were associated with better care experience perceptions, whereas spouse (β = −5.03, *p* = .00) and adult child (β = −5.88, *p* = .00) caregivers, higher anticipatory grief scores (β = −0.08, *p* = .00), older adult functional dependency (β = −0.20, *p* = .02), and verbally aggressive agitation behaviors (β = −1.50, *p* = .01) were associated with worse.

## Discussion

Decedent data from the PISCES cohort illuminates the last year of life with dementia for community-dwelling Singaporeans and their caregivers. We find many of the same clinical symptoms and healthcare utilization patterns in our Singaporean older adult sample as previously documented among older adults in western long-term care settings; however, the involvement and influence of informal caregivers differentiate our findings from prior studies. Informal family caregivers played a major role in older adults’ final year, contributing the equivalent of more than a full-time job or S$30 000 in caregiving annually and influencing EOL care patterns.

Community-dwelling older adults experienced many of the clinical complications and distressing symptoms described among older adults living in nursing homes in prior studies—pneumonia, febrile episodes, and UTIs—which were associated with increased medical intervention.^6^^,^^34^ Potentially burdensome interventions, many of questionable value, were in proportion to or more widespread than previously described.^6^^,^^35^ Nearly half of older adults received one or more antimicrobial, consistent with prior studies documenting poor adherence with the minimal clinical criteria for antimicrobial initiation.^6^ Almost a quarter of older adults were tube-fed, a much higher proportion than most western contexts, and higher than prior findings among non-decedents in Singapore, as more than half of tube feeding was initiated in the final year.^11^^,^^34^ Clinical guidance discourages tube feeding among older adults, recommending careful hand feeding instead. Nevertheless, prior qualitative findings suggest cultural values around filial piety, fears, and clinician recommendations drive utilization in Singapore.^11^ Tube feeding in turn fuels the use of physical restraints to prevent older adults from removing tubes.^12^ The current study suggests restraint use is even more widespread than previously documented, experienced by 70% of decedents, and initiated even late in life despite relatively low levels of physical agitation, often associated with restraint use.^12^

Nearly half of older adults experienced an inpatient hospital admission, higher than most prior studies conducted among older adults living in long-term care facilities, putting older adults at risk for often taxing and costly care experiences shown to have limited clinical benefit.^36^ Moreover, preliminary bereavement data indicates admissions continued beyond the final survey point, with more than one-third of older adults dying in hospital, a much higher proportion than in the United States (10%).^37^ UTIs were the strongest predictor of inpatient admissions, consistent with recent findings from the United States showing older adults with dementia are twice as likely as those without dementia to be diagnosed with a UTI in the emergency department despite a lower symptom prevalence.^38^^,^^39^

In addition to clinical burdens, caregiver preferences and perceptions were shown to have a significant influence on EOL care patterns. Caregivers who preferred comfort care only were much more likely to have loved ones with admissions but less likely to report medical interventions. These findings likely reflect the culture of strong filial piety in which caregivers may feel compelled to do all they can to reduce suffering for their loved ones, seeking hospital care as needed to promote these goals.^13^ This is consistent with prior findings in EOL cancer care in Singapore in which most admissions in the last year of life were for maintenance or comfort care rather than high-intensity interventions.^40^ However, nearly one-third of caregivers indicated prolonging life as a care goal, which likely also suggests the importance of filial values, as most who indicated this care goal were children of the older adult. A recent Delphi study to develop a multidimensional international palliative care goals model specific to dementia failed to reach consensus on whether the goal of prolonging life should be included in the model.^41^ Though controversial for the panel, prolonging life appears to be a common and influential care goal among Singaporean caregivers in our study, potentially highlighting a point of resistance to a palliative approach embedded in the culture.^41^

Finally, this study elucidates the extraordinary burden faced by family caregivers. Less than 10% of decedents spent time in nursing homes in their final year, suggesting the burden of care was largely borne by cohabiting family members, many of whom were employed and providing care to other relatives. Indeed, 30% of caregivers quit their job to take care of the older adult in their final year, devoting over 40 hours per week. Even though most caregivers were supported by a domestic helper, this did not significantly lessen caregiving hours. Many caregivers were dissatisfied with the older adult’s care experience, particularly if the caregiver was struggling with anticipatory grief, their relative was severely dependent and demonstrated verbally aggressive agitation behaviors, suggesting these needs were not sufficiently addressed. These findings along with the pervasive use of restraints attest to the multifaceted burden of dementia caregiving and inadequate caregiver support. Our findings also lend credence to conclusions from a recent systematic review of home-based EOL dementia care suggesting the intense level of support required as dementia progresses may surpass the capacity of home environments, even with dedicated informal caregiver involvement.^42^

### Clinical and policy implications

This study highlights the persistence of hospital admissions and potentially burdensome interventions among community-dwelling older adults in the last year of life and an insufficient supporting environment to facilitate patient-centered EOL dementia care in the community.^17^^,^^43^^,^^44^ Potentially burdensome interventions are common in the hospital setting,^35^ but their ubiquity in the last year of life has not been previously documented in the context of dementia among community-dwelling older adults.^45^ In advanced dementia, a palliative approach should guide all interactions with the healthcare system and advise care at home to alleviate symptoms and minimize burden for older adults and their caregivers. Although some strategies, such as provider education, audit and feedback, and medication review have been effective in reducing low-value care, such as antipsychotic prescriptions among older adults in nursing home settings, new strategies are required to reduce low-value care among community-dwelling older adults, who experience similar clinical burdens and potentially more burdensome interventions.^46^ Moreover, community-based strategies must be cognizant of cultural norms and values such as filial piety, which may contradict a palliative approach and consider caregiver education to promote a palliative approach. Likewise, in many Asian contexts such as Singapore that lack robust long-term care infrastructure, additional policy supports beyond Singapore’s existing “Home Caregiving” grants—which offer up to S$4800 annually to offset informal caregiving costs, far less than the average S$30 000 incurred—are needed to bridge the gap to support dementia caregivers in the community and enable a palliative approach.

### Limitations

This study benefits from rich longitudinal data but is not without limitations. The PISCES cohort of caregivers to community-dwelling older adults was recruited from memory/geriatrics clinics and general medicine wards of 7 major public hospitals, 6 home care foundations, and 2 hospices across Singapore, but a national-level sampling frame was not used. We rely on caregiver-reported data, which is subject to recall and cognitive bias and may not accurately reflect older adult outcomes such as quality of life and pain. We cannot differentiate by dementia type or cause of death. Additionally, our study may underestimate interventions and healthcare utilization, because caregivers only report on the prior 4 months and 63 caregivers did not complete all 3 surveys in the older adult’s final year. Likewise, the preliminary bereavement data finds 35% of older adults died in hospital, suggesting additional inpatient admissions following the last survey point.

### Conclusions

Our study highlights the unique dementia EOL experiences of community-dwelling older adults in Singapore. Our findings suggest the need for a palliative approach, which is currently not apparent in the high levels of potentially burdensome intervention and acute care utilization observed, nor consistent with caregiver values related to prolonging life. As the number of older adults grows with increased reliance on family caregivers at home, much work is needed to support caregivers in providing a palliative approach and ensuring they can capably care for their relatives without reliance on low-value, harmful interventions.

## Supplementary Material

glaf227_Supplementary_Data
