# Supplementary material for: The final year for community-dwelling older adults with dementia in an Asian setting: admissions, interventions, and caregiver burden
Source: J Gerontol A Biol Sci Med Sci. 2025 Nov 16;80(12):glaf227. doi: 10.1093/gerona/glaf227 (PMC12672495; doi:10.1093/gerona/glaf227)
Supplement: glaf227_Supplementary_Data [file glaf227_supplementary_data.docx]

Supplementary materials

eFigure 1. Flowchart showing process for obtaining analytical sample


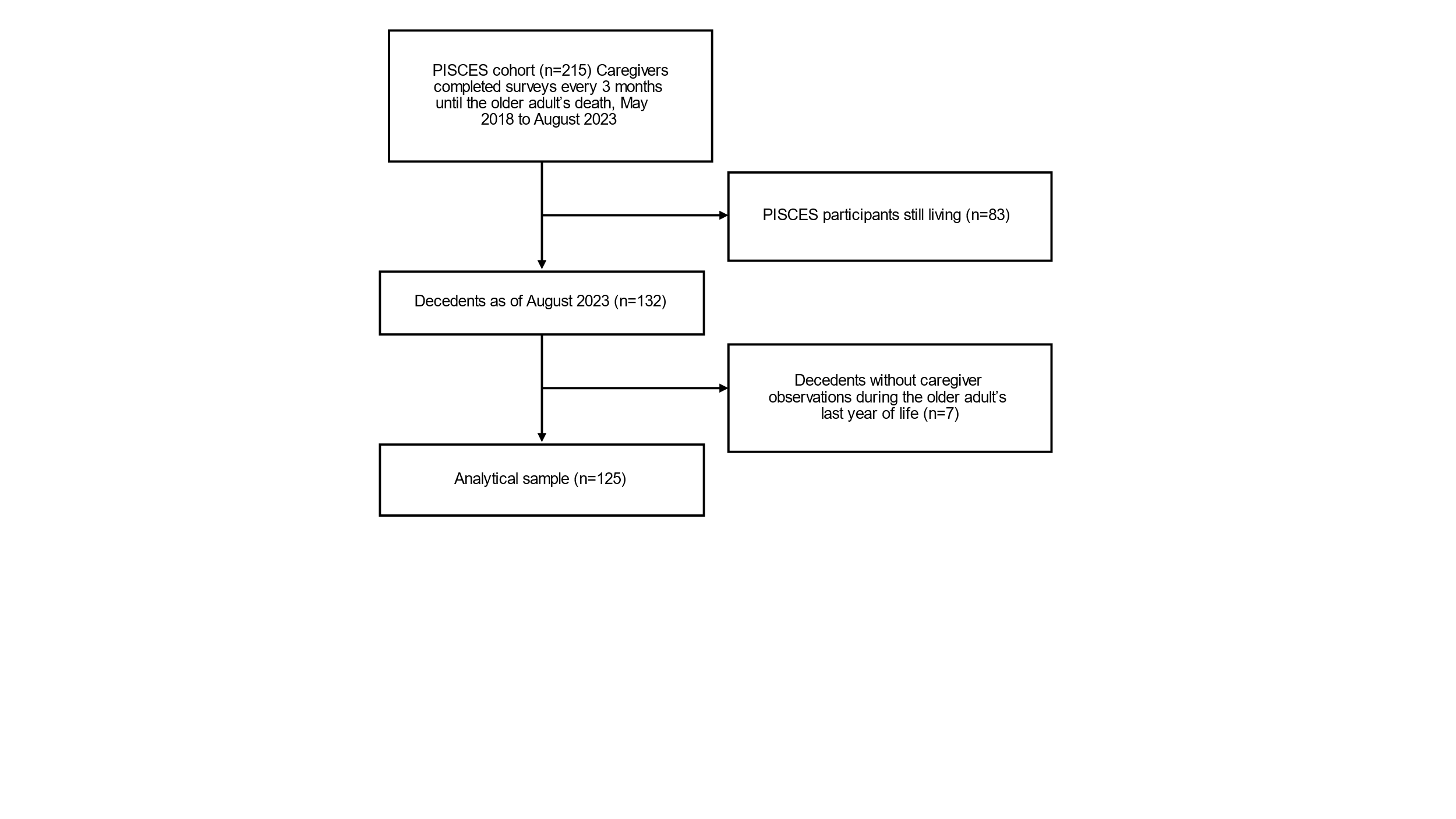


eTable 1. Mean informal caregiving hours and estimated annual value

| **Types of caregiving** | **Mean hours/ week** | **Annual value in Singapore Dollars**  [S$ 1∼ US$ 0.77] | | |
| --- | --- | --- | --- | --- |
|  |  | **Median** | **25^th^ percentile** | **75^th^ percentile** |
| ADLs* | 16.2 | S$12,441 | S$11,003 | S$15,761 |
| IADLs^†^ | 13.7 | S$10,536 | S$9,319 | S$13,348 |
| Supervision | 23.6 | S$18,176 | S$16,077 | S$23,027 |
| ADLS & IADLS | 29.9 | S$22,977 | S$20,322 | S$29,109 |
| Total (all 3) | 41.7 | S$32,125 | S$28,414 | S$40,700 |

***Activities of daily living (ADLs), ^†^instrumental ADLs (IADLs)
